# Chronic Cold Exposure Leads to Daytime Preference in the Circadian Expression of Hepatic Metabolic Genes

**DOI:** 10.3389/fphys.2022.865627

**Published:** 2022-05-17

**Authors:** Zhida Zhang, Le Cheng, Junxian Ma, Xiaomei Wang, Yingying Zhao

**Affiliations:** ^1^ BGI College & Henan Institute of Medical and Pharmaceutical Sciences, Zhengzhou University, Zhengzhou, China; ^2^ BGI -Yunnan, BGI - Shenzhen, Kunming, China; ^3^ Department of Physiology, School of Basic Medical Sciences, Shenzhen University Health Sciences Center, Shenzhen, China

**Keywords:** circadian rhythm, metabolism, liver, chronic cold, adaption

## Abstract

Circadian control allows organisms to anticipate and adapt to environmental changes through changes in physiology and behavior. The circadian system timing is entrained by cues, such as light, food, and temperature. An ambient temperature dramatically impacts the sleep–wake cycle and metabolic rhythmicity. As endotherms, mammals rely on tissues such as the liver to provide fuel for thermogenesis to maintain body temperature. The adaptive response of the circadian rhythm of liver metabolism to chronic cold exposure remains largely unexplored. Here, we investigated the circadian rhythm adaptation of hepatic metabolism in response to environmental cold stress using a mouse model of chronic cold exposure. We analyzed metabolites and transcripts of mouse livers at 24 h and found that long-term low-temperature exposure resulted in a synergistic and phase synchronization of transcriptional rhythms of many genes associated with metabolic pathways. Notably, transcription peaked in the early light phase when the body temperature was relatively low. Our results suggest that chronic cold does not alter the rhythmic expression of essential core clock genes in the liver, so the rewiring of clock control gene expression is another mechanism that optimizes the circadian rhythm of liver metabolism to meet the energy requirements of animal thermogenesis.

## Introduction

Circadian rhythms are physiological and behavioral changes that follow a 24-h cycle. The circadian clock effectively orchestrates adaptations in behavior and physiology in response to fluctuations in environmental conditions. Many genes, functioning in the metabolism of nutrients, xenobiotics, and production of bioactive molecules, are under circadian regulation (Panda et al., 2002). The circadian system is entrained by timing cues, such as light, food, and temperature (Reppert and Weaver, 2002). Numerous studies have demonstrated that the circadian clock is a central regulator of energy metabolism. The diurnal oscillation of metabolism controlled by the circadian clock system anticipates and prepares for periodic and predictable changes. Disruption of circadian rhythmicity can induce metabolic disorders such as obesity and diabetes (Takahashi et al., 2008; Bass and Takahashi, 2010; Bass and Lazar, 2016; Adlanmerini et al., 2019).

As endotherms, mammals consume energy to maintain body temperature. An ambient temperature significantly affects sleep–wake cycles and metabolic rhythms (Gerhart-Hines et al., 2013; Panda, 2016; Machado et al., 2018; Adlanmerini et al., 2019). It has been reported that the core clock protein Per2 plays an essential role in adaptation to low temperatures (Chappuis et al., 2013), and the nuclear receptor Rev-erbα controls circadian thermogenic plasticity (Gerhart-Hines et al., 2013; Adlanmerini et al., 2019). Body temperature regulation in response to cold is controlled by thermogenic brown adipose tissue (BAT); consequently, most studies have focused on entrainment of circadian rhythms and circadian clocks in mouse BAT by chronic cold (Chappuis et al., 2013; Gerhart-Hines et al., 2013; Machado et al., 2018; Adlanmerini et al., 2019). However, the liver plays a central role in nutrient metabolism, with circadian rhythms detected in the expression of more than 25% of liver transcripts (Panda, 2016; Patke et al., 2020). The rhythmic gene expression in the liver provides energy-saving benefits and allows temporal separation of incompatible biochemical reactions that regulate the rate-limiting metabolic processes (Panda, 2016). Despite a substantial body of evidence demonstrating the impact of ambient temperature on the rhythmicity of organ metabolism, little is known about the challenges to hepatic glucose and lipid metabolism and metabolic rhythmicity and the mechanism underlying adaptation to chronic cold in tissues, such as the liver.

Our study adopted an omics approach to study the impact of chronic cold exposure on the circadian rhythms and circadian clock in mouse liver. Chronic cold exposure can reset the liver metabolome, change the rhythmic characteristics of the transcriptome, and make the expression of a large number of genes more rhythmic. We observed that chronic cold stress altered the rhythmicity of glucose metabolism, fatty acid β-oxidation, and thermogenesis. Our findings suggest rhythmic-concerted effect and phase synchronization of liver metabolism as an energy-saving strategy for chronic cold exposure.

## Materials and Methods

Mice. All animal studies were performed under protocols approved by the Laboratory Animal Ethics Committee of Shenzhen University. All experiments were carried out on 10- to 12-week-old (20–25 g) littermates. Mice were group-housed in a specific-pathogen-free and temperature- and humidity-controlled animal facility (the temperature was 22°C, and the relative humidity was 50–60%), under a 12:12-h light–dark cycle (lights on at 7 a.m. [defined as zeitgeber time zero (ZT0)] and lights off at 7 p.m.). Mice were allowed free access to food and water throughout the experiment.

Wild-type C57BL/6J male mice were obtained from the Guangdong Medical Laboratory Animal Center (Guangzhou, China) [SPF, SCXK (Yue) 2018–0002, Guangzhou, China]. The mice were randomly divided into the following two groups (*n* = 48 per group): the control group and the chronic cold group (CC group), each of which was divided into six-time points (*n* = 8 per time point). The PER2-luciferase mice were obtained from the Jackson Laboratory. The PER2-luciferase mice were randomly divided into the following groups (*n* = 8 per group): the control and chronic cold groups (CC groups).

### Cold Exposure and Anal Temperature Measurements

Before 4-C exposure, the mice were kept at 18°C for 1 week to acclimate to the environment. Mice acclimated to 18°C were placed in 4–5°C precooled cages with cotton, free food, and water and exposed to the cold for 1 week, with corresponding experiments performed at six-time points on the eighth day. (Emmett et al., 2017). During cold exposure, the anal temperature of mice was measured using a thermometer at ZT0 and ZT12 h. The mice were removed from the study and euthanized if the anal temperature fell ≥10°C from the average measurement.

### *In Vivo* Monitoring of Peripheral PER2::LUC Rhythms

PER2:Luc mice were subjected to *in vivo* fluorescence assay by intraperitoneal injection of fluorescein (15 mg/kg body weight) at corresponding time points on day eight after 1 week of chronic cold exposure (Tahara et al., 2012). The bioluminescence values of mice were determined 8 minutes after fluorescein injection using a small animal fluorescence imaging system. On a 24-h cycle, the luminescence of the PER2 protein was determined every 4 h.

### Locomotor Rhythms Recording

The animal video tracking software Ethovision XT (Noldus, Wageningen, Netherlands) was used to record and analyze the locomotor behavior of the mice. Ten-week-old male C57BL/6 mice (*n* = 4) were selected for observation and recording. Spontaneous motor activity is defined as the distance traveled per unit of time (three min). After the mice adapted to the observation cage, they were divided into experimental and control groups and given different experimental conditions to record continuously for 1 week.

### Oil Red O Staining and H&E Staining Analyses

A portion of liver tissue was quickly frozen by immersing it in the Tissue-Tek OCT compound. The frozen tissue was cut into 10-µm sections and stained with Oil Red O solution at room temperature. The tissue specimens were sealed with glycerin gelatin. A part of the tissue was fixed with paraformaldehyde, dehydrated, and embedded with paraffin, and the tissue was cut into 5-µm sections for H&E staining. Histopathological sections were examined using a Nikon Ti-E fluorescence microscope (Nikon, Tokyo, Japan).

### RNA-Seq Data Analysis

We collected liver tissue (ZT3, ZT7, ZT11, ZT15, ZT19, and ZT23) every 4 h within 24 h and collected liver samples of experimental and control mice (*n* = 18 per group and *n* = 3 each time point). Total RNA was extracted from tissues using TRIzol (Invitrogen, Carlsbad, CA, United States). The quantity and quality of total RNA were determined using the Nano Drop and Agilent 2100 bioanalyzer (Thermo Fisher Scientific, MA, United States). The total RNA was enriched by the mRNA enrichment method to construct a DNA library; the library quality was checked on the Agilent Technologies 2100 bioanalyzer. The final library was formatted after being qualified. The final amplified DNA nanosphere (DNB) was loaded into the pattern nanoarray with Phi29, and the peer-end 100-base reading was generated on the BGIseq500 platform (BGI-Shenzhen, China).

The JTK_CYCLE algorithm (Hughes et al., 2010) was used to identify the periodic characteristics of each group. The delta value and period are 4 and 24, respectively, which can detect periodic features with a 12–28 h period, with an “independent” method. Based on a 24-h oscillation period (*p* < 0.05), transcripts were divided into two categories: rhythmic genes (JTK algorithm, *p* < 0.05) and non-rhythmic genes (JTK algorithm, *p* ≥ 0.05). Other parameters were set to default. The JTK_CYCLE algorithm determined the genes with rhythmic change, and the nonlinear cosine regression tool CircaCompare (Parsons et al., 2020) was used to detect amplitude (i.e., half the difference between the peak and trough of a given response variable), MESOR (the midline statistic for estimating rhythms), and acrophase (the time at which the response variable peaks) rhythm characteristics. The differences between the experimental group and the control group were compared.

### Time-Series Clustering Analysis

We conducted noise-robust soft cluster analysis on each time series using the Mfuzz package’s fuzzy c-means clustering method (Futschik, 2015). We set the core threshold to automated evaluation, clusters to four, and all other settings to default in the R package.

### Untargeted Metabolomics Analysis

Liver samples from experimental mice were collected (*n* = 12 per group and *n* = 6 each time point). Untargeted metabolomics analysis was performed by BGI (Shenzhen, China). Metabolites from liver tissues were isolated and processed for quality control samples. Metabolites were separated and detected using a Waters 2D UPLC (Waters, United States) and a Thermo Fisher Scientific Q Exactive HF high-resolution mass spectrometer (Thermo Fisher Scientific, United States). A BEH C18 column (1.7 m 2.1*100 mm, Waters, United States) analyzed positive and negative ion modes. A Q Exactive HF mass spectrometer (Thermo Fisher Scientific, United States) was used for primary and secondary mass spectrometry data caused. Mass spectrometry scan determined that the plasma–nucleus ratio ranges from 70 to 1,050, the first-level resolution is 120,000, the AGC is 3E6, and the maximum injection duration is 100 ms (IT, injection time). Compound Discoverer 3.1 (Thermo Fisher Scientific, United States) software processed the LC–MS/MS data. It entails the extraction of peaks, the alignment of peaks, and the identification of compounds. For data preprocessing, statistical analysis, metabolite classification labeling, and functional annotation, the R software package metaX (Wen et al., 2017) and the metabolite information analysis program were utilized. The PENN (Di Guida et al., 2016) method was used to normalize the data to obtain the relative peak area. QC-RLSC (Dunn et al., 2011) was used to correct the batch effect, and compounds with CV (coefficient of variation) greater than 30% of the relative peak area were eliminated. PCA was used to reduce the dimensionality of the original multivariable data, examine variable grouping within the data set, and identify similarity, difference, and outliers within and between sample groups. To screen for differential metabolites, the VIP values of the first two principal components of the PLSPLS-DA (partial least squares method-discriminant analysis) (Barker and Rayens, 2003; Westerhuis et al., 2008) model were merged with the univariate fold change and Student’s *t*-test findings. Screening conditions for differential metabolites are as follows: ①the first two principal components of the PLS-DA model VIP≥1, ②fold-change ≥1.2 or ≤0.83, ③*p*-value<0.05. Quantitative and qualitative analysis of compounds was conducted to obtain the confidence level, and the KEGG pathway annotation and enrichment were performed.

## Results

### Chronic Cold Alters the Circadian Rhythms and Metabolism in Mouse Liver

**FIGURE 1:**
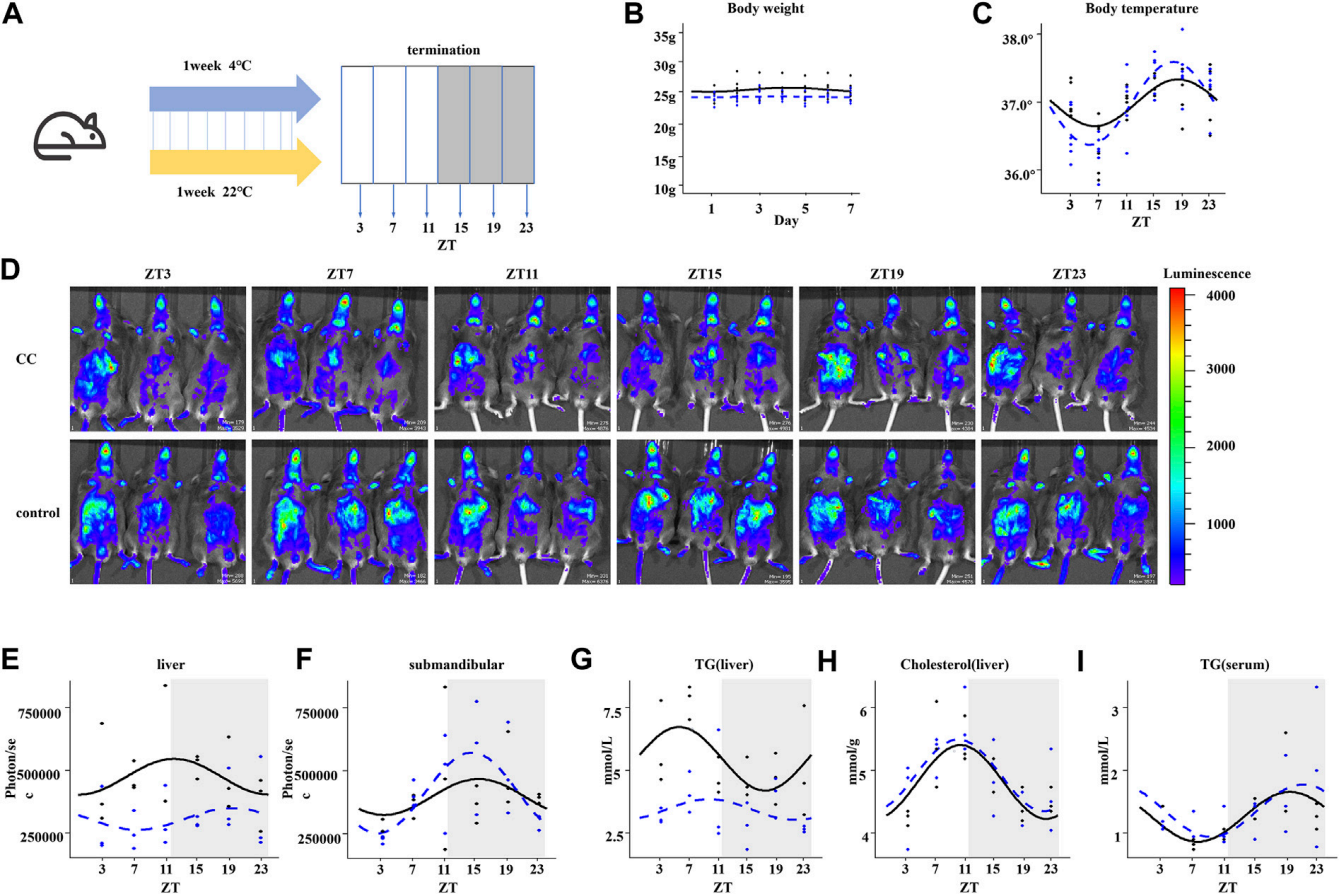
Chronic cold alters the circadian rhythms and metabolism in mouse liver. **(A)** Workflow of the chronic cold exposure experiment in mice. After 1 week of WT mice housed at 22°C or 4°C, the corresponding indexes were measured, or tissues and organs were collected at 4-h intervals within 24 h. **(B)** Body weight of WT mice. **(C)** Body temperature of WT mice. The black line indicates the control group, and the blue dotted line indicates the chronic cold (CC) group; points are individual data points, and lines represent cosinor regression fit; the body temperature rhythm between control and CC groups was compared; the amplitude was significantly different; the *p*-value was 0.028 (<0.05). **(D)** Fluorescent images of PER2: LUC mice. At each time point, three PER2: LUC mice were detected simultaneously. The fluorescence of the liver and submandibular gland eight min after injection of fluorescein is shown . **(E)** Quantification of the liver bioluminescence data in **(D)**, in photons per second, showed a significant difference in MESOR between CC and control groups. *p*-value was 0.0004 (less than 0.05), and there was no statistical significance in other rhythm characteristics. **(F)** Quantification of the submandibular gland bioluminescence data in **(D)**, in photons per second. **(G)** Circadian liver triglyceride content from WT mice between the control and CC groups; the MESOR was significantly different; the *p*-value was 0.00005 (<0.05). **(H)** Circadian liver cholesterol. **(I)** Circadian serum cholesterol. The black line indicates the control group, and the blue dotted line indicates the chronic cold (CC) group. Points are individual data points, and lines represent cosinor regression fit.

To investigate the influence of chronic cold exposure on the circadian rhythmicity of hepatic metabolism, C57BL/6J mice were housed for 1 week at 4°C or 22°C in regular 12:12-h light–dark conditions and with free access to food and water (Figure 1A). We observed a slight decrease in the body weight of mice in the chronic cold exposure (CC) group compared with those in the control group (Figure 1B). CC mice maintained a 24-h rhythmicity of body temperature but with increased amplitude (Figure 1C). We then investigated the circadian rhythms of clock gene expression in the peripheral organs of mice by non-invasive fluorescence imaging of the liver and submandibular glands of PER2:LUC mice eight min after injection with the fluorescent marker (Tahara et al., 2012). During chronic cold exposure, the expression rhythm of PER2 protein in the liver was changed, mainly as a decrease in MESOR (midline statistics of rhythm estimation), with no significant difference in phase change (Figures 1D,E). In contrast, chronic cold exposure did not significantly impact the rhythmicity of PER2 protein levels in the submandibular gland (Figure 1F). Since the liver plays a pivotal role in energy supply, we examined the rhythmicity of hepatic triglyceride (TG) and cholesterol levels. The diurnal profile of hepatic TG levels was rhythmic, with a peak at ZT7 and a trough at approximately ZT19 in the control group. Interestingly, chronic cold exposure induced an arrhythmic pattern and an overall dampening of TG levels in the liver of mice (Figure 1G). The 24-h circadian rhythms of hepatic-free cholesterol were maintained in CC mice (Figure 1H), and there was no difference between the rhythmic profiles of serum TG levels of the CC and control groups (Figure 1I). These results suggested that chronic cold exposure affects the circadian rhythmicity of mouse liver metabolism, with marked changes in response to the whole-body energy demand. We next performed transcriptomic and untargeted metabolomics studies to identify altered key metabolites and their functions.

### Chronic Cold Exposure Alters the Hepatic Metabolome

**FIGURE 2:**
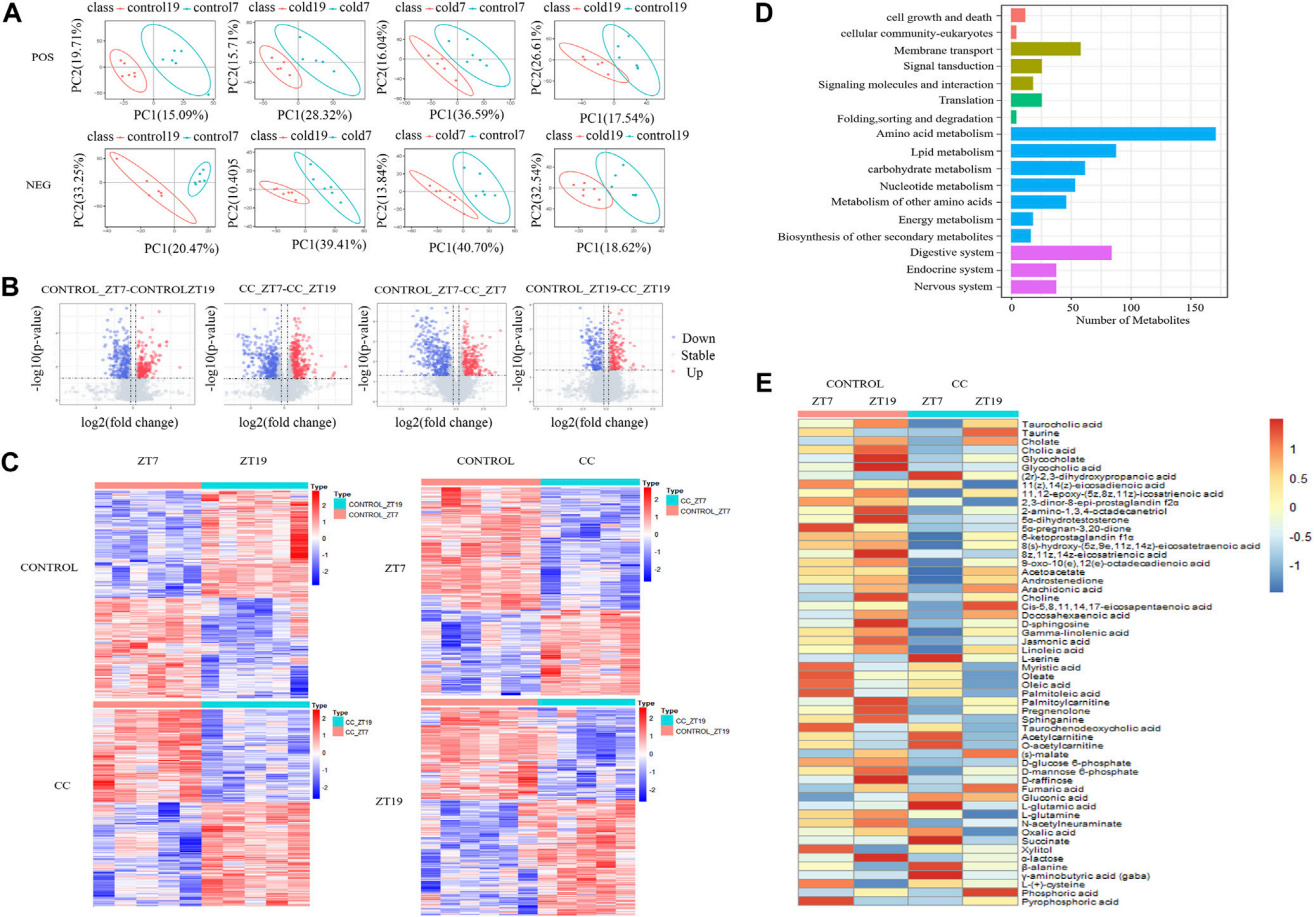
Chronic cold exposure alters the hepatic metabolome. **(A)** PLS-DA score plots from the control and chronic cold (CC) groups, control ZT7-control ZT19 in positive mode (R^2^ = 0.98, Q^2^ = 0.47) and negative mode (R^2^ = 0.99, Q^2^ = 0.83); CC ZT7-CC ZT19 in positive mode (R^2^ = 0.97, Q^2^ = 0.51) and negative mode (R^2^ = 0.99, Q^2^ = 0.69); control ZT7-CC ZT7 in positive mode (R^2^ = 0.99, Q^2^ = 0.59) and negative mode (R^2^ = 0.98, Q^2^ = 0.56); control ZT19-CC ZT19 in positive mode (R^2^ = 0.98, Q^2^ = 0.18) and negative mode (R^2^ = 0.96, Q^2^ = 0.4). **(B)** Volcano maps for comparing differential metabolites between the control group and the CC group and between ZT7 and ZT19 time points, upregulated metabolites (red dots) and downregulated metabolites (blue dots), and identification of differential metabolites (VIP≥1, fold-change ≥1.2 or ≤0.83, and *p* < 0.05). **(C)** Heat maps show the differential metabolites at ZT7 and ZT19 under control and CC conditions. The expression level is expressed in the heat map as the Z-score. Each sample is represented by a column, and each metabolite is represented by a line. **(D)** KEGG functional annotation bar chart depicts the metabolites that differ between the ZT7 and ZT19 time points under control and CC circumstances. **(E)** Heat map depicting the distribution of all diurnal oscillatory metabolites in the liver. Intensity levels are represented as the Z-score-scaled means (*n* = 5/6 group).

For our untargeted metabolomics analysis, partial least squares discriminant analysis (PLS-DA) (Barker and Rayens, 2003; Westerhuis et al., 2008) was used to evaluate the difference between the CC and control groups at two times, i.e., midday ZT7 and midnight ZT19. As shown in Figure 2A, all four groups demonstrated excellent stability (*R* ^2^ ≥ 0.96) in both the positive and negative ion modes, and the majority of groups had an excellent prediction effect (Q^2^ > 0.5). By combining positive and negative ion models, we compared the differential metabolites at ZT7 and ZT19 time points in the control group according to screening conditions. We found 663 differentially expressed metabolites in the control group; comparing ZT7 and ZT19 time points revealed 980 differentially expressed metabolites in the CC group. In the photoperiod ZT7, there were 742 differential metabolites between the control and CC groups, while in the dark phase ZT19, there were 446 differential metabolites between the control and CC groups (Figure 2B). There were 1,188 differential metabolites between the control and CC groups at two-time points (Figure 2C). The differential metabolites were quantitatively and qualitatively analyzed, and the KEGG database was used to classify the identified differential metabolites by functional annotation (Figure 2D). As expected, differential metabolites were mainly concentrated in nutrient metabolism and nutrient transport. A comparison of the hepatic levels of 84 metabolites related to lipid, carbohydrate, and energy metabolism between the control and CC groups revealed that malate and fumaric acids share a similar pattern with increased levels at ZT19 in the CC group (Figure 2E). Interestingly, succinic acid and l-serine exhibited a similar pattern in the control group, remaining at similar levels between ZT7 and ZT19, while ZT7 levels were significantly elevated in the CC group. Taurine showed reversal, with low levels in the light phase and high levels in the dark phase. The carbohydrate and energy metabolism metabolites in the CC group were significantly changed, and the level of glutamic acid was significantly increased in the light period. In addition, we observed that chronic cold exposure caused many differential metabolites to have similar change patterns, such as taurine, taurocholate, bile salts, cholate, glycocholate, and glycolic acid.

The majority of the lipid metabolism-related compounds in the CC group were present at lower levels than those in the control group. The saturated fatty acid myristic acid and the unsaturated fatty acids oleic acid and palmitoleic acid exhibited similar patterns of variation, with relatively low levels at ZT7 in the CC group. However, the polyunsaturated fatty acids arachidonic acid and docosahexaenoic acid exhibited similar variation patterns, with little change under CC stress. Palmitoylcarnitine concentration fell in the CC group, and it was much lower at ZT7 than at ZT19. Acetylcarnitine and O-acetylcarnitine exhibited the same variation trend, and the difference between ZT7 and ZT19 was more significant under chronic cold conditions. Compared with the control group, the levels of acetylcarnitine were higher at ZT7 and lower at ZT19. In contrast, acetoacetic acid, which is essential for forming ketone bodies by β-oxidized fatty acids (Laffel, 1999), had lower levels at ZT7 and higher levels at ZT19 in the CC group. Our results suggest that the changes of hepatic metabolites are an adaptation to chronic cold exposure.

### Chronic Cold Exposure Alters the Circadian Rhythm of Transcriptional Gene Expression in the Liver

**FIGURE 3:**
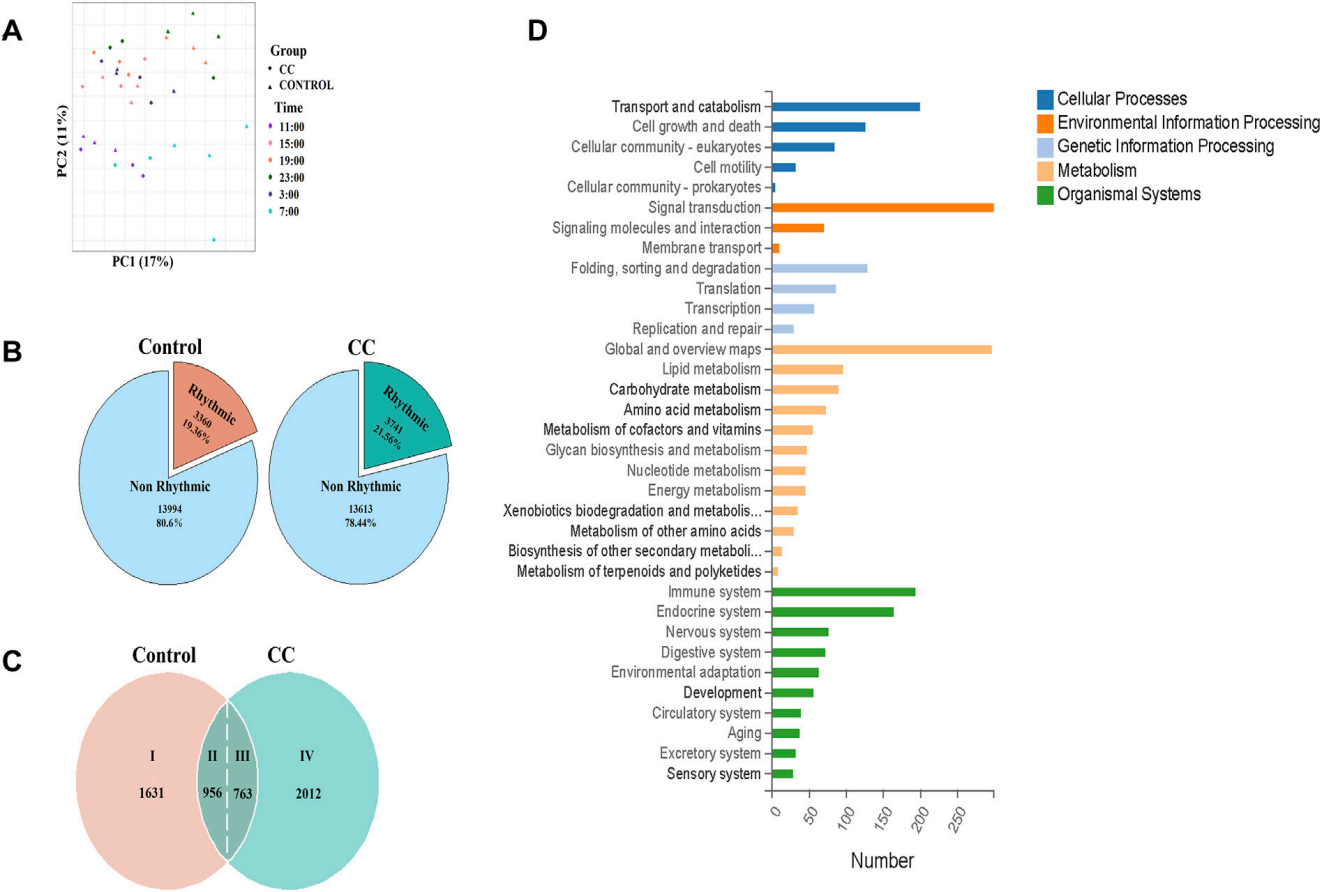
Chronic cold exposure alters the circadian rhythm of transcriptional gene expression in the liver. **(A)** Principal component analysis (PCA) of samples based on liver genes, with color indicating sampling time. The circle indicates the control group, and the triangle indicates the chronic cold (CC) group. **(B)** Pie chart of chronic cold (CC) transcriptome composition for the control group. **(C)** Rhythmic transcriptome was classified based on the expression pattern of each gene under each environment and genotype—the pie chart illustrating the distribution of genes in the liver by category. **(D)** KEGG functional annotation bar chart depicts the function of 2775 genes classified as category Ⅲ and Ⅳ groups.

To evaluate the effect of chronic cold on mouse liver transcriptome oscillations, we conducted a transcriptomic analysis of liver tissues collected every 4 h for a 24-h period (ZT3, ZT7, ZT11, ZT15, ZT19, and ZT23). Principal component analysis exhibited that under the dual influence of different time and experimental treatments, most of the liver transcripts of the samples indicated the formation of intra-group aggregation and inter-group dispersion, with minor intra-group differences and significant inter-group differences, which was suitable for further analysis (Figure 3A). In Figure 3B, rhythmic genes accounted for about one-fifth of all genes in the control and CC groups. Compared with those of the control group, the proportion of rhythmic genes in the CC group was 2.2% high, and 381 more genes showed oscillatory characteristics. In chronobiological studies, circadian rhythms are evaluated in terms of amplitude (i.e., half the difference between the peak and trough of a given response variable), phase (the time at which the response variable peaks), and MESOR (the midline statistic for estimating rhythms) (Kubo et al., 2012; Lee et al., 2012). We further analyzed the rhythmicity of the oscillatory genes in both CC and control groups using CircaCompare (Parsons et al., 2020). Compared with the control group, chronic cold changed the expression pattern of a large number of genes. As illustrated in Figure 3C, we identified four groups of genes: (I) 1,631 oscillatory genes lost rhythmicity under chronic cold exposure; (II) 956 oscillatory genes maintained their original rhythmicity under chronic cold exposure; (III) 763 oscillatory genes exhibited altered rhythmic characteristics under chronic cold exposure; and (IV) 2,012 oscillatory genes gained rhythmicity under chronic cold exposure. KEGG analysis of the genes in groups III and IV revealed that 384 genes functioned in the various metabolic pathways, including 96 genes linked to lipid metabolism, 90 genes related to carbohydrate metabolism, and 45 genes associated with energy metabolism (Figure 3D). KEGG enrichment analysis exhibited that the genes in groups III and IV were significantly enriched in metabolic pathways induced by changes in external temperature (Supplementary Figure S1A). GO enrichment analysis also showed a similar situation. The genes in groups III and IV were enriched in lipid metabolism, the process of oxidation–reduction, and phosphorylation (Supplementary Figure S1A).

### Temporal Analysis Revealed That Chronic Cold Induced Changes in Specific Transcription Patterns

**FIGURE 4:**
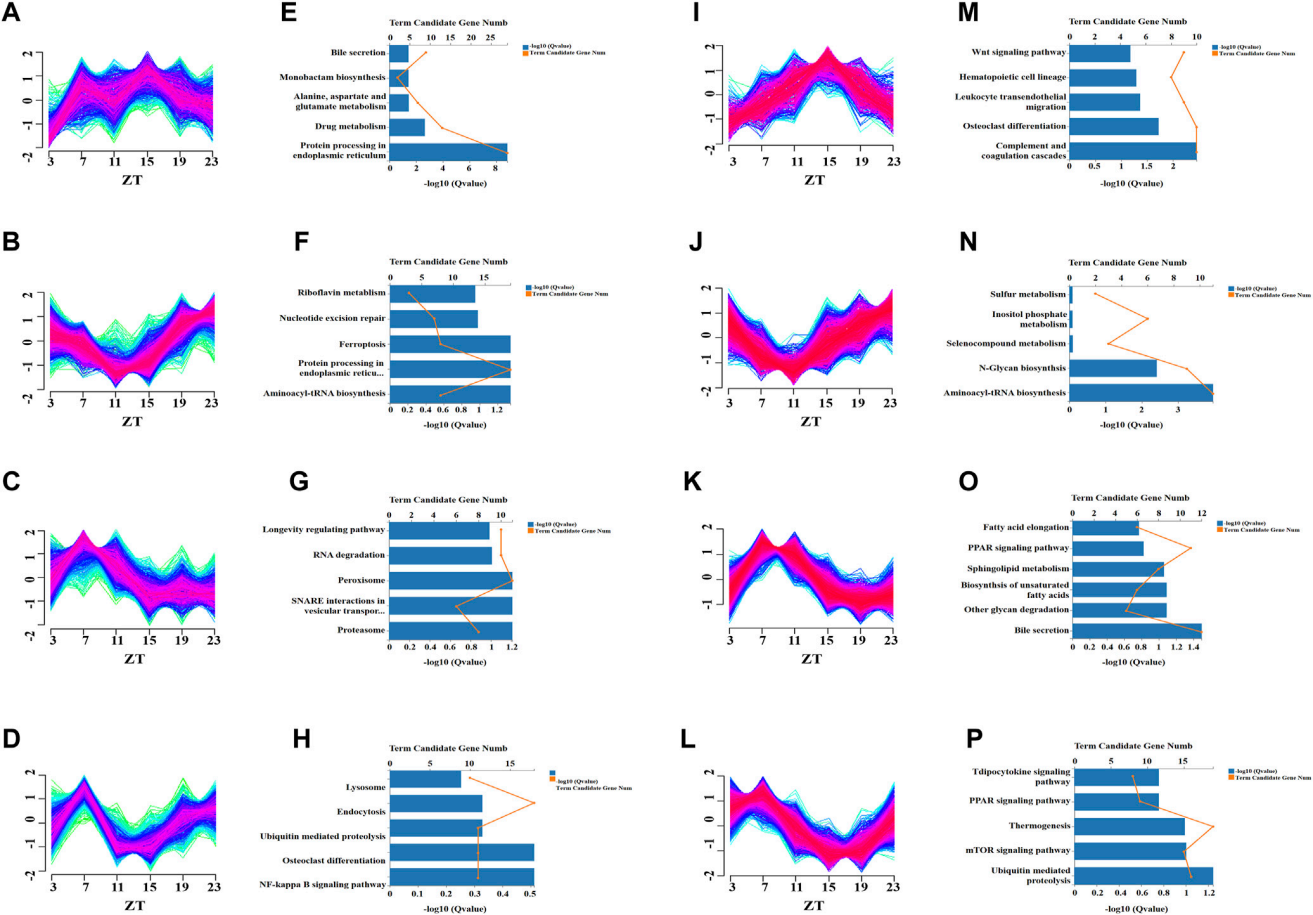
Temporal analysis revealed that chronic cold-induced changes in specific transcription patterns. Clustering analysis was performed on the genes of Ⅲ and Ⅳ liver groups (from Figure 3C), and the control and chronic cold groups were separated into four different enrichment clusters. **(A–D)** Temporal gene Z-scores from four different enriched clusters to the control group of the liver. The expression values were standardized. The blue and green lines represent low-membership-value genes, while the red lines represent high-membership-value genes. **(E–H)** KEGG pathway enrichment analysis for the transcripts in clusters 1–4 unique to the control group of the liver (*p* < 0.05). **(I–L)** Temporal gene Z-scores from four different enriched clusters of the liver’s chronic cold (CC) group. **(M–P)** KEGG pathway enrichment analysis for the transcripts in clusters 1–4 of the chronic cold (CC) group of the liver (*p* < 0.05).

In time-series analysis, expression patterns of genes related to time changes can be presented, and genes with similar expression patterns will be grouped into the same cluster (Futschik, 2015). We used time-series analysis to determine how chronic cold changes the overall expression pattern of rhythmic genes and those with similar expression patterns. The global profiling of CC-induced rhythmic genes (groups III and IV) was evaluated by clustering and KEGG pathway enrichment analyses. During the 24-h cycle, the rhythmically expressed genes were divided into four independent oscillatory clusters according to different expression patterns in the control group (Figures 4A–H) and CC group (Figures 4I–P), and the corresponding KEGG enrichment analysis was performed. The expression patterns of cluster 1 in control and CC groups were different (Figures 4A,I), and the main KEGG enrichment pathways differed (Figures 4E,M). The expression pattern of cluster 2 genes between control and CC groups indicated that the time of peak and trough appeared was different (Figures 4B,J), but they were significantly enriched in the amino-tRNA biosynthesis pathway (Figures 4F,N). In cluster 3, the expression patterns of the control and CC groups’ expression patterns were similar (Figures 4C,K), but the main enrichment pathways of KEGG were significantly different. The CC group was mainly enriched in bile acid secretion, polysaccharide degradation, unsaturated fatty acid synthesis, PPAR signal transduction, and fatty acid extension (Figures 4G,O). These results suggested that CC profoundly reshaped the expression of lipid metabolism genes. In cluster 4, there were significant differences in expression patterns between the control group and the CC group (Figures 4D,L). In addition, the main enrichment pathways were different between the two groups, while the CC group was mainly enriched in the mTOR signaling pathway, thermogenesis, and PPAR signaling pathway (Figures 4O,P). Thus, long-term cold exposure apparently altered the cluster-dependent transcriptional and expression patterns of metabolism-related rhythmic genes.

### Chronic Cold Exposure Triggers the Synchronization of Oscillatory Metabolic Gene Expression

**FIGURE 5:**
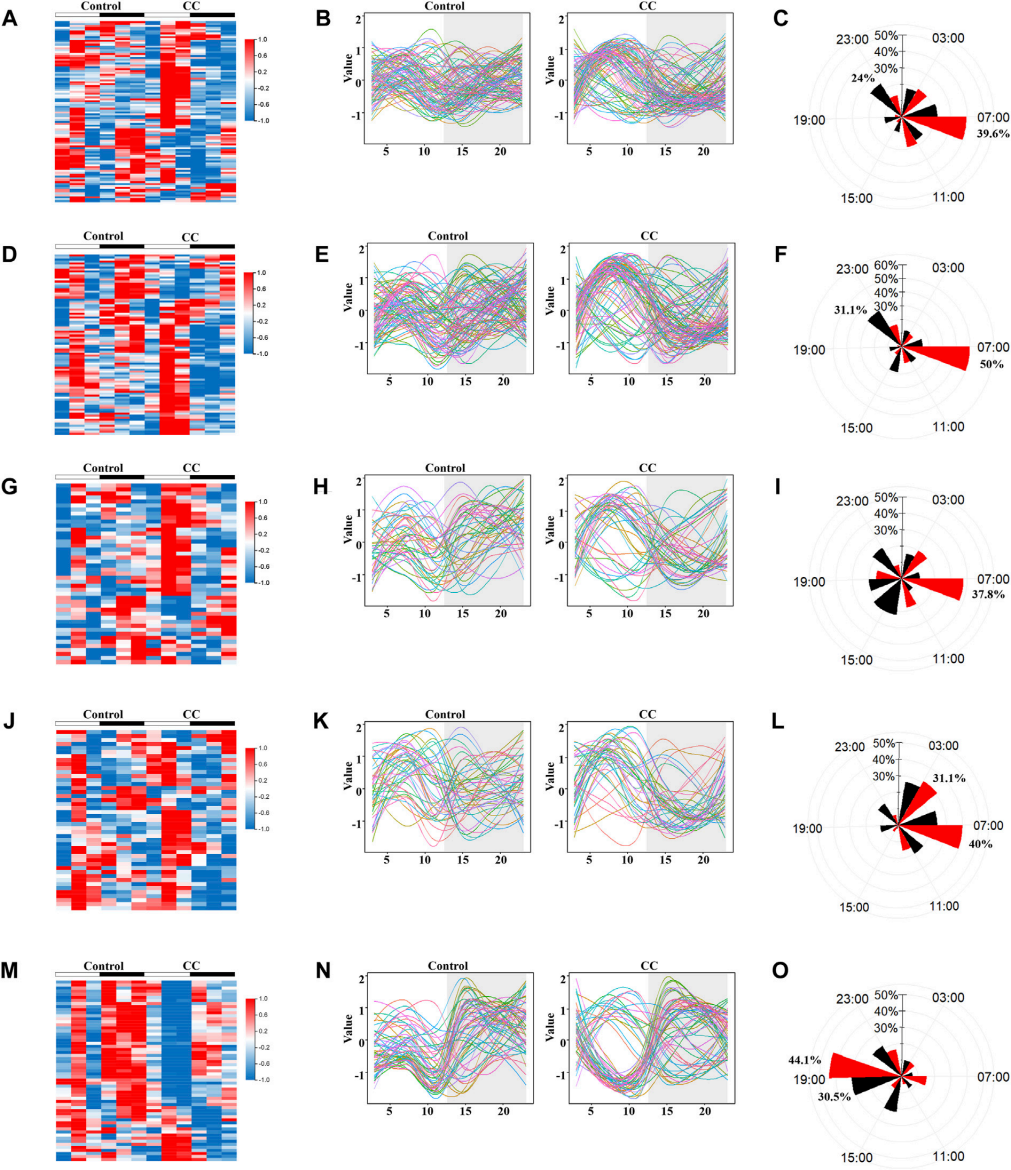
Chronic cold exposure triggers the synchronization of oscillatory metabolic gene expression. KEGG pathway enrichment analysis was performed on the Ⅲ and Ⅳ liver group genes (Figure 3C), and genes related to metabolic pathways were selected for rhythm analysis. Heat map of genes enriched in control and chronic cold (CC) groups; color represents Z-score-normalized expression; each sample is represented by a column, and a line represents each metabolite. Each line in the fitted curve represents a gene, with expression levels represented as a Z-score of the mean (*n* = 3), random colors, and time points on the horizontal axis (ZT). The radar plots of acrophase with a black line representing the control group, and a red line representing the chronic cold group. **(A–C)** Lipid metabolism genes **(D–F)**, carbohydrate metabolism genes, **(G–I)** energy metabolism genes, **(J–L)** thermogenesis genes, and **(M–O)** the protein processing in endoplasmic reticulum genes.

To explore the influence of prolonged external cold stress on the rhythmicity of liver metabolism, genes relevant to lipid, carbohydrate, energy metabolism, and thermogenesis were grouped based on metabolome data. Heat maps revealed substantial circadian shifts in the expression of genes involved in lipid, carbohydrate, energy metabolism, and thermogenesis (Figures 5A,D,G,J,, respectively). Interestingly, most of the gene phases shifted tended to be concentrated at ZT7, with peak expression in this phase (Figures 5B,E,H,K). To determine whether rhythmicity resetting is a universal phenomenon and also occurs in non-metabolic pathways, we evaluated the genes enriched in the protein processing in ER pathways with the lowest Q value according to the KEGG pathway enrichment analysis. Like the metabolic routes, rhythmic profiling of gene functioning ER protein processing was also synchronized but peaked at ZT19, with troughs at ZT10. CircaCompare analysis revealed higher amplitude after chronic cold exposure, but without statistical significance, with a subtle change in MESOR (Supplementary Figures S2A–E). The expression of each gene is visualized in Figures 5B,E,H,K, N following Z-score function normalization and fitting. In Figure 5B, it can be seen that a considerable number of genes related to lipid metabolism in both the control group and the CC group peaked during the day, but the CC group had more peaks, and the trend was more pronounced. Carbohydrate metabolism-related genes peaked at night in the control group and peaked in the daytime in the CC group (Figure 5E). In addition, most of the lipid metabolism and carbohydrate metabolism-related genes in the CC group increased gradually at night, which made their amplitudes more pronounced. The phase distributions of genes related to energy metabolism and thermogenesis in the CC group were also concentrated in the light phase (Figures 5H,K); on the contrary, the phases of protein processing in endoplasmic reticulum-related genes were in the dark phase in both the control and CC groups (Figure 5N). A detailed analysis of gene phase distribution exhibited that in the CC group, lipid, carbohydrate, energy metabolism, and thermogenesis gene phases accounted for 39.6, 50, 37.8, and 40% of the total at ZT7 (Figures 5C,F,I,L, respectively). The lipid and carbohydrate metabolism gene phases were the most numerous in the control group at ZT23 (24 and 31.1%, respectively). Most thermogenic genes were expressed in photoperiod due to chronic cold, and more than 70% of thermogenic genes reached their peak values at Z73 and ZT7 (Figure 5L). The genes phases of protein processing in the ER pathway accounted for the most significant proportion in the control and CC groups at ZT19 (30.5 and 44.1%, respectively) (Figure 5O). Thus, these findings suggest that chronic cold exposure induces phase shifts in a significant number of genes involved in lipid, carbohydrate, energy metabolism, and thermogenesis which peaked in the light phase.

### Chronic Cold Exposure Synchronizes the Expression of a Large Number of Metabolic Genes

**FIGURE 6:**
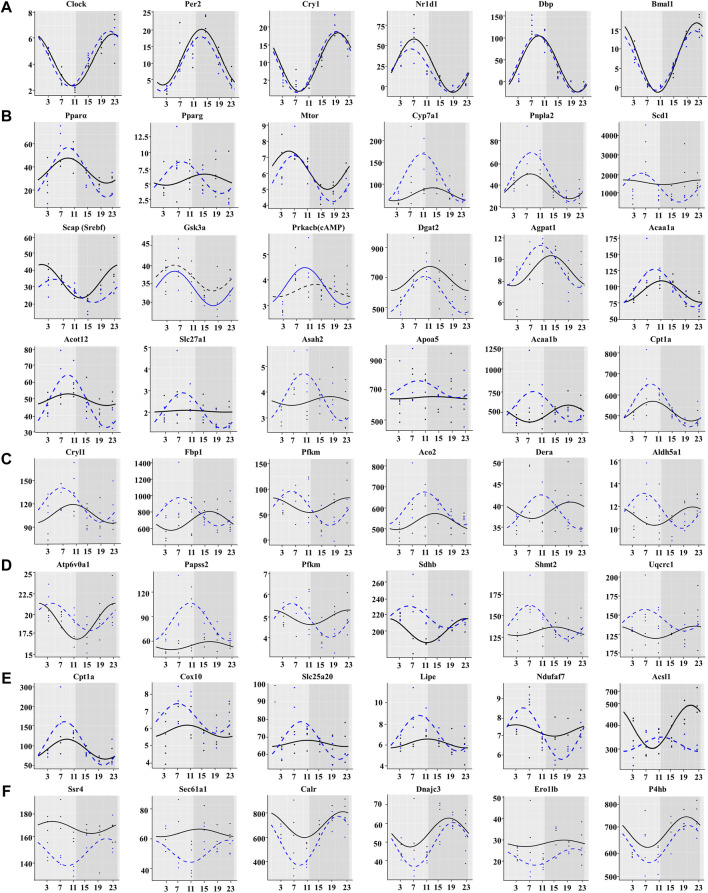
Chronic cold exposure synchronizes the expression of a large number of metabolic genes. **(A)** Expression of core clock genes, **(B)** lipid metabolism genes, **(C)** carbohydrate metabolism genes, **(D)** energy metabolism genes, **(E)** thermogenesis genes, and **(F)** protein processing in endoplasmic reticulum genes. The black line indicates the control group, and the blue dotted line indicates the chronic cold (CC) group. Points are individual data points, and lines represent cosinor regression fit.

As expected, the chronic cold did not alter the expression of the core circadian genes, including Clock, Bmal1, Per2, Cry1, Nr1d2, and Dbp (Figure 6A). We did not observe significant differences in the characteristic rhythm MESOR, amplitude, or acrophase of these core rhythm genes between the two groups (Figure 6A). However, chronic cold significantly altered the rhythmic expression of lipid metabolism-related genes, with shifts in the phases of many lipid metabolism-related genes, peaking synchronously around ZT7 (Figure 6B). The rhythmic expression of beta-oxidation-related genes, such as *Cpt1a*, *Slc22a5*, *Slc22a4*, *Cpt2*, and *Acad9*, was reset in the CC group. The amplitude of the *PPARα* phase, an important regulator of lipid metabolism (Bougarne et al., 2018), was significantly increased. *PPARγ* and *Apoa5* expression levels switched from non-rhythmic to rhythmic. *Acaa1b* and *Prkacb(cAMP)* genes showed changes in amplitude, phase, and MESOR. The amplitude of the *Cyp7a1*, *Asah2*, *Slc27a1*, and *Acot12* phases was elevated considerably. The oscillatory expression patterns of genes involved in carbohydrate and energy metabolism exhibited similar patterns of changes, with the majority peaking between photoperiods ZT7 and ZT11. The phases of *Dera*, *Sdhb*, and *Uqcrcl* were reversed, and the amplitude and MESOR of *Fbp1*, *Aco2*, *Papss2*, and *Shmt2* were significantly altered (Figures 6C,D). Such rhythmic changes also occurred in genes related to thermogenesis. Most of the genes reached their peak at photoperiod, *Acsl1* rhythmic expression was reset by day and night, and *Ndufaf7* changed from arrhythmic to rhythmic (Figure 6E). We further analyzed CC-induced expression patterns of genes related to ER protein processing. Interestingly, the rhythmicity of these genes was also reset but with synchronized peaks in the dark photoperiod (Figure 6F).

## Discussion

We investigated the diurnal adaptation of liver metabolism under chronic cold stress. Chronic cold exposure can reset the liver metabolome and alter the rhythmic characteristics of the transcriptome. The ambient temperature change is related to the synchronization of the phase of rhythmic genes in the liver metabolic pathway. Additionally, we demonstrated that persistent cold stress alters the rhythms of glucose metabolism, fatty acid β-oxidation, and thermogenesis. The circadian plasticity of liver metabolism allows for adaptive changes in multiple metabolic pathways, resulting in synergistic effects to optimize heat source energy supply during long-term low-temperature exposure.

The effects of ambient temperature on the rhythm of liver metabolism were investigated in this study. After chronic cold exposure, the body weight of mice decreased slightly, but there was no significant difference. The amplitude of temperature change between day and night was enlarged, indicating that chronic cold exposure had an enormous burden on maintaining the body temperature of mice. We observed changes in the rhythm expression pattern of the core clock protein PER2, a decrease in liver PER2 protein content, and changes in the rhythm signature MESOR. Per2 is related to adaptation to low-temperature environments in animals, and it regulates adaptive thermogenesis through Ucp1 activity and transcription (Chappuis et al., 2013). TG levels in the liver of mice were dramatically lowered following long-term exposure to cold, and there was no significant circadian regularity, as in prior research (Bartelt et al., 2011). Following previous research (Tokizawa et al., 2009), our observations suggest that an adaptive circadian metabolic response occurs under cold stress conditions.

To understand diurnal response of the hepatic metabolome to chronic cold exposure, we analyzed diurnal changes in the metabolites and transcriptomes of mouse liver tissues. The results indicate that adaptation to liver dynamics involves remodeling of the metabolic response to chronic cold exposure. The liver plays a central role in the glucose metabolism, maintaining relatively stable levels in the blood, with the enzyme glucose-6-phosphatase produced playing a key role. Chronic cold exposure leads to relatively low levels of glucose-6-phosphate, suggesting that glucose is continuously consumed as a significant energy source in the liver. However, glutamic acid levels were significantly elevated at ZT7 and reduced at ZT19. These findings suggest that gluconeogenesis is active at ZT7 (Aikawa et al., 1972), corresponding to when the lowest body temperature occurs during daytime rest in nocturnal rodents.

Chronic cold in mice triggers adaptive thermogenesis, a process mediated by brown fat and assisted by the liver (Adlanmerini et al., 2019). Brown fat cells need to increase their intake of carbohydrates and triglycerides and cholesterol. This process requires the liver to convert cholesterol into bile acids through an alternative synthetic pathway and relies on cytochrome P450 (Worthmann et al., 2017). The liver is the only organ that converts cholesterol to bile acids (Norlin and Wikvall, 2007). The changes of taurine at ZT7 and ZT19 were utterly opposed to those in the control group, with low light phase content and high dark phase content, which might relate to the rhythmicity of bile acid secretion (Chesney, 1985). More than 10 cytochrome P450s and related genes are reported to be under circadian regulation (Panda et al., 2002). Interestingly, our investigation discovered that the gene rhythms of over ten cytochrome P450 families represented by Cyp7a1 and Cyp4a14 changed, and the cycle of Cyp7a1 changed significantly after chronic cold exposure, with higher amplitude and 4-h phase shift. The increase of l-serine in photophase ZT7 may be related to the decrease of TG accumulation (Sim et al., 2015), which is burned to provide heat under cold conditions. Additionally, cold-induced thermogenesis induces a metabolic switch in the liver, resulting in the production of acylcarnitine, which fuels brown fat thermogenesis (Simcox et al., 2017). This adequately explains the changes in the metabolomes of palmitoylcarnitine, acetylcarnitine, and o-acetylcarnitine. We found that acetoacetate levels reached relatively low levels in the photophase under chronic cold exposure, related to the increase of β-oxidation during daytime. Acetoacetate is a crucial ketone body produced from the β-oxidation of fatty acids and released into the bloodstream from the liver as an important energy source (Laffel, 1999). Our results suggest that changes in liver metabolite content can provide a better fuel supply for animals to adapt to extreme temperature conditions.

Chronic cold profoundly reshaped the rhythms of the mouse liver transcriptome, yet it did not alter the central clock mechanism. The chronic cold caused changes in rhythm characteristics in more than two-thirds of rhythm genes when other conditions were equal, such as light exposure and diet. Many genes of III and IV groups were enriched into metabolism-related pathways. It suggests that mice have an adaptive rhythm change in metabolism. It is more intuitive that the rhythm genes of thermogenesis pathways in mice that adapt to the chronic cold environment also have adaptive changes. Genes represented by Acsl1 have a 12-h phase reversal to achieve heat production and maintain body temperature. In time-series analysis, genes with similar expression patterns are grouped into the same cluster (Futschik, 2015). The investigation revealed that genes related to lipid metabolism, PPAR signaling pathway, and bile secretion were grouped into a cluster. PPAR signaling pathway, thermogenesis, mTOR signaling pathway, and other related genes were divided under the same cluster, and the expression patterns of the two clusters were similar. This suggests that these metabolic and thermogenic rhythm genes have rhythm parameters changed and tend to have similar expression patterns.

**FIGURE 7:**
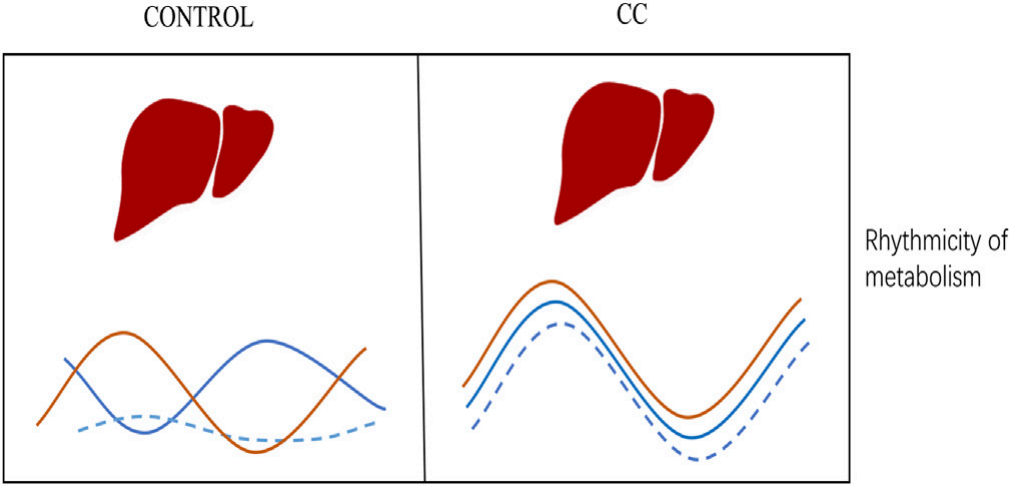
Summary model encapsulates the most significant findings. A schematic diagram may explain differences in gene rhythm expression caused by chronic cold conditions (see details in the text).

Based on this, we further observed that the phases of rhythm genes related to lipid metabolism, carbohydrate metabolism, energy metabolism, and thermogenesis tended to be synchronized, and a large number of them reached the peak at ZT7 during the photoperiod. In particular, more than 70% of the rhythmical genes related to thermogenesis reached their peak at ZT3 and ZT7. Therefore, we believe that chronic cold reconstructs the rhythmic expression of many genes. The expression patterns of many genes involved in metabolism and thermogenesis were modulated from arrhythmia to rhythmicity, and the amplitude, MESOR, and phase of many genes were altered. Many genes were in in-phase synchronization, and the peak appeared mainly in photoperiod ZT7. These changes optimize energy supply to maintain body temperature for nocturnal animals at rest during the day (Figure 7).

In addition to its effect on metabolism, chronic cold induced synchronization of expression of genes related to ER protein processing. In contrast, the expression of these genes appeared to peak in the dark, reversing the metabolic tendency. We hypothesize that optimization of energy expenditure is the dominant factor that determines the benefit of changes in the circadian rhythm. In the early morning, the majority of energy is used to maintain body temperature, and hence the timing of other cellular events, such as protein processing, are reset during the hours of darkness. This resetting of metabolic rhythmicity allows animals to compensate for changes in the nutrient and energy supply to maintain body temperature.

Circadian clocks have fundamental roles in maintaining liver homeostasis by regulating processess such as energy metabolism and the expression of enzymes involved in nutrient absorption and metabolism (Tahara and Shibata, 2016). Although chronic cold has a significant impact on metabolic rhythm, it was found in the transcriptome that chronic cold exposure did not disrupt the rhythmic expression of some critical core clock genes, such as *Clock, Per2, Cry1,* and *Dbp.* It may be that the rhythms of the central clock genes remain unchanged because our feeding patterns and light conditions remain the same. The study by Masri et al (2016) also found no changes in the expression of liver clock genes but changes in the expression of liver clock control genes, which may be other clues that cause this change, which remains to be explored. In addition, to ensure mice’s health and energy supply during the experiment, mice were free to obtain food, which may lead to changes in the eating time and frequency in the CC and control groups. Although such changes did not change the expression rhythm of core rhythm genes, they might affect the metabolome results; further research needs to be noted. Consistent with a previous study, *Fasn*, *Acc*, *Acly*, and other genes involved in *de novo* lipogenesis were expressed at a lower level in the liver during prolonged cold (Adlanmerini et al., 2019). The mechanism by which the liver inhibits *de novo* lipogenesis synthesis in a cold environment is not clear.

In this study, we provide evidence that the liver has an important physiological role in long-term adaptation to chronic cold exposure. Our results suggest that chronic cold provides an adaptive response to the high energy requirements of thermogenesis by inducing the resetting of metabolic rhythms in the liver through a synergistic effect of metabolic genes. We are actively exploring mechanisms to regulate this metabolic rhythm adaptation. The regulation of the metabolic rhythm in the liver is necessary for long-term adaptation to cold, and the discovery of this regulation feature provides strategies for improving metabolic disorders.

## Data Availability

The datasets presented in this study can be found in online repositories. The names of the repository/repositories and accession number(s) can be found below: The transcriptome sequencing raw data is stored in the NCBI database with the project number ID PRJNA 802697. Metabolome data generated in this study are available in the MetaboLights database under the project number MTBLS4248.
