# Supplementary figures and images for: Chronic Cold Exposure Leads to Daytime Preference in the Circadian Expression of Hepatic Metabolic Genes
Source: Front Physiol. 2022 May 17;13:865627. doi: 10.3389/fphys.2022.865627 (PMC9152247; doi:10.3389/fphys.2022.865627)

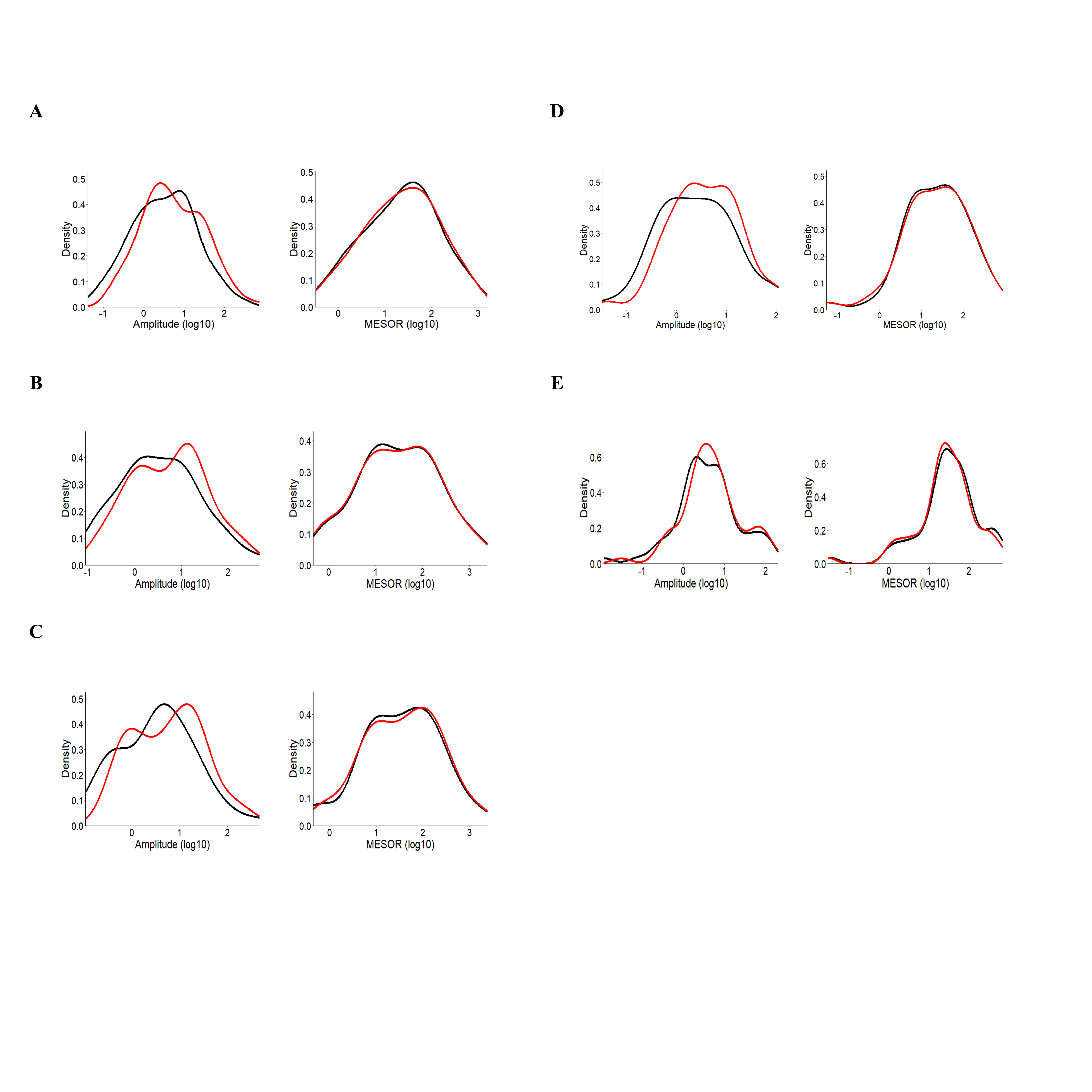

Supplement: Supplementary file 1 [file Image2.TIF]

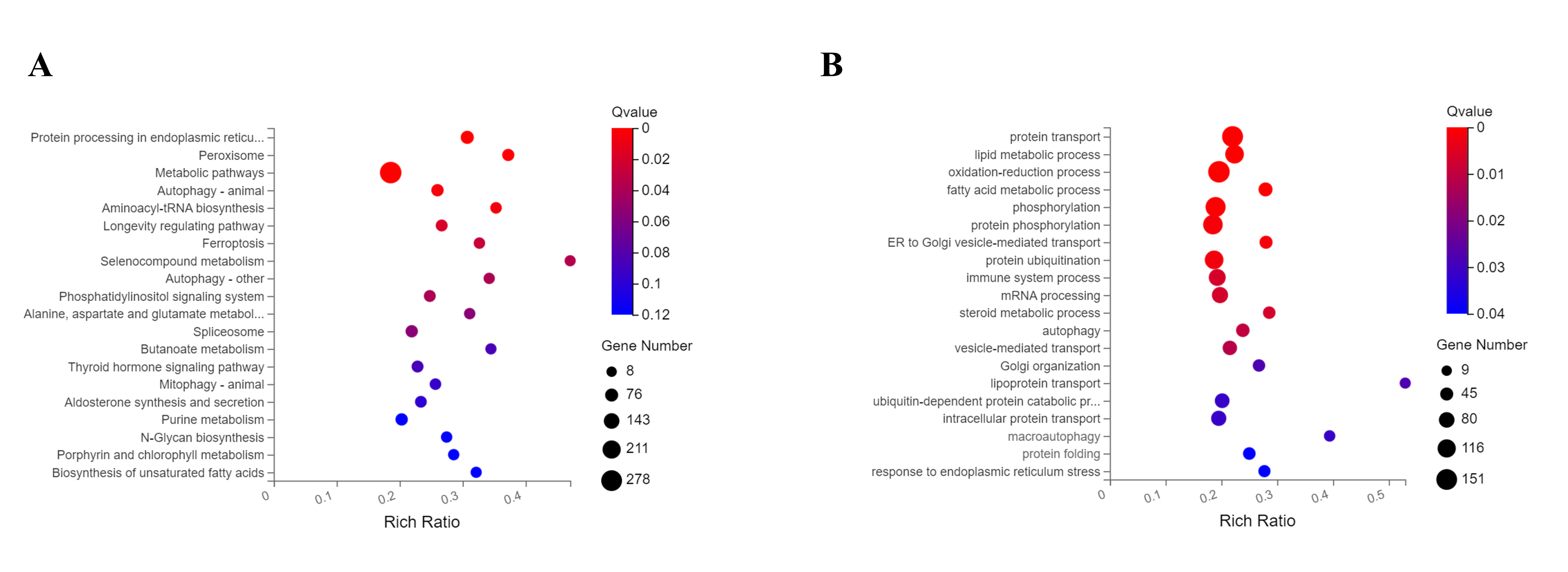

Supplement: Supplementary file 2 [file Image1.TIF]
